# Neutrophilic airways inflammation in lung cancer: the role of exhaled LTB-4 and IL-8

**DOI:** 10.1186/1471-2407-11-226

**Published:** 2011-06-07

**Authors:** Giovanna E Carpagnano, Grazia P Palladino, Donato Lacedonia, Anna Koutelou, Silvio Orlando, Maria P Foschino-Barbaro

**Affiliations:** 1 Department of Medical and Occupational Sciences, Institute of Respiratory Disease, University of Foggia, Via degli Aviatori 1, Foggia, 71100, Italy; 2 Department of Thoracic Surgery, Casa di Cura La Madonnina, viale Pasteur 18, Bari, 70124, Italy

**Keywords:** lung cancer, LTB-4, IL-8, non-invasive methods, breath cancer, airways inflammation, neutrophils

## Abstract

**Background:**

Recent advances in lung cancer biology presuppose its inflammatory origin. In this regard, LTB-4 and IL-8 are recognized to play a crucial role in neutrophil recruitment into airways during lung cancer.

Notwithstanding the intriguing hypothesis, the exact role of neutrophilic inflammation in tumour biology remains complex and not completely known.

The aim of this study was to give our contribution in this field by investigating LTB-4 and IL-8 in the breath condensate of NSCLC patients and verifying their role in cancer development and progression.

**Method:**

We enrolled 50 NSCLC patients and 35 controls. LTB-4 and IL-8 concentrations were measured in the breath condensate and the blood of all the subjects under study using EIA kits. Thirty NSCLC patients and ten controls underwent induced sputum collection and analysis.

**Results:**

LTB-4 and IL-8 resulted higher in breath condensate and the blood of NSCLC patients compared to controls. Significantly higher concentrations were found as the cancer stages progressed. A positive correlation was observed between exhaled IL-8 and LTB-4 and the percentage of neutrophils in the induced sputum.

**Conclusion:**

The high concentrations of exhaled LTB-4 and IL-8 showed the presence of a neutrophilic inflammation in the airways of NSCLC patients and gave a further support to the inflammatory signalling in lung cancer. These exhaled proteins could represent a suitable non-invasive marker in the diagnosis and monitoring of lung cancer.

## Background

Recent advances in cancer biology point to a role of inflammatory signalling in lung cancer and encourage the reconsideration of the diagnostic and prognostic value of inflammatory markers.

Currently there is a large number of clinical data confirming the inflammatory origin of lung cancer: 1) in the lung, chronic inflammatory diseases such as sarcoidosis, fibrosis or chronic obstructive pulmonary disease (COPD) have been associated with a higher risk of cancer development [1-7]; 2) several studies demonstrated that human lung-cancer risk can be modified by polymorphisms in pro-inflammatory genes such as interleukin (IL)-1 beta and the tumor necrosis factor (TNF)α [8,9]; 3) other studies showed that long-term users of anti-inflammatory drugs may be at a reduced risk of cancer development [10].

The inflammatory processes in the lung are characterized by the influx of neutrophils into the airways [11,12]. The migration and activation of leucocytes within the airways may be the consequence of soluble proteins released by resident cells that are activated by various stimuli. Leukotriene (LTB)-4 and IL-8 are recognized to play a crucial role in neutrophil recruitment into airways during lung cancer [13,14].

LTB-4 is a lipoxygenase product of arachidonic acid metabolism, released primarily by polymorphonuclear and mononuclear phagocytes as well as by epithelial cells [15-18]. LTB-4 is usually used as an indicator of the state of activation of neutrophils [19]. LTB-4 concentrations are high in blood and pleural effusion [19] but have not as yet been measured in other airway samples of lung cancer patients.

Interleukin-8 (IL-8), one of the ELR+ CXC families of chemokines, is a potent pro-angiogenic factor and furthermore, its expression is associated with angiogenesis, tumour progression and survival in patients with non-small cell lung cancer (NSCLC) [20-27]. Several studies in blood and airways (broncho-alveolar lavage, pleural effusion, tumour tissue) supported its role as marker of lung cancer [19,22,28-31]. Recently higher levels of IL-8 have been described, also in the exhaled breath condensate (EBC) of NSCLC patients [32].

An increasing interest has been recently generated among non-invasive methods that sample airways by the collection of EBC, often more used for the biological study of lung diseases, such as cancer [33]. The complete non-invasiveness of this method makes the latter more readily accepted by patients, such as those affected by cancer, who are likely to already be exhausted, not only by their condition, but also by the huge number of exams they have had to undergo. Although the study of tumour markers in exhaled breath condensate did not reach the clinical settings, except in asthma and COPD, a good number of studies are readily available in literature and propose the use of this sample for the non-invasive diagnosis and monitoring of lung cancer [28,32,34-36].

The aim of the present study was to give further support to the neutrophilic inflammatory signalling in lung cancer analysing the LTB-4 and the IL-8 in the EBC of NSCLC patients and controls.

## Methods

### Characteristics of the Patients

50 consecutive patients affected by non-small cell lung cancer and 35 control subjects (without pulmonary diseases) who consented to the study, were enrolled at the Unit of Thoracic Surgery, Casa di cura La Madonnina, Bari, and at the Department of Respiratory Disease, Foggia University (Table 1). Written informed consent was obtained from all subjects upon approval of the study by the Ethic Committees of the two hospitals.

**Table 1:** Demographic and clinical data of subjects enrolled

	NSCLC patients	healthy subjects
N	50	35
sex (M/F)	36/14	18/17
AGE (yr)	65 ± 7	64 ± 1
FEV1	98.7 ± 3.4	101 ± 3.3
FVC	100 ± 3.2	102.1 ± 2.7
Hystotype:		
squamous cell carcinoma	22	-
adenocarcinoma	28	-
Stage:		
I	13	-
II	12	-
III	8	-
IV	17	-
Smoking habit:		
current smokers	25	10
ex-smokers	15	-
non-smokers	10	25

All the patients were enrolled in the study immediately before histological diagnosis. Furthermore, none of them had received any form of anti-cancer therapy, invasive diagnostic procedure or primary lung surgery. Following cytohistological diagnosis, patients with cancer underwent standard staging procedures consisting of a physical examination, serum chemistry analysis, brain, chest and abdomen CT scans, radionuclide bone scan, and bronchoscopy. The diagnosis of NSCLC was done either by bronchoscopic biopsy or by transthoracic needle aspiration. Squamous cell carcinoma was diagnosed in twenty-two subjects, whereas the remaining subjects received a cytohistological diagnosis of adenocarcinoma. Overall, NSCLC patients were classified as stage I in 13 cases, stage II in 12 cases, stage III in 8 cases and stage IV in 17 cases.

All of the patients enrolled underwent exhaled breath condensate (EBC) and whole-blood (WB) collection at enrolment. The day after the subjects underwent induced sputum collection and analysis.

We acquired information on their smoking habits and classified the smokers (according to the number of pack-years smoked) into two groups: mild (group 1 [patients]: <40 pack-years) and heavy smokers (group 2 [patients]: >40 pack-years). Twenty-five of the lung cancer patients were current smokers (54 ± 8 pack/year); fifteen were ex-smokers (44 ± 6 pack/year) and had quit at least three years before, whereas ten were non-smokers. Ten healthy subjects were smokers while the remaining ones were non-smokers.

### EBC and WB collection and processing

1 mL of EBC and 3 mL of WB in one setting from each patient.

The EBC was collected by using a condenser, which allowed for the non-invasive collection of non-gaseous components of the expiratory air (EcoScreen Jaeger, Wurzburg, Germany). The condensate was collected in ice at -20°C, transferred to 1,5 ml polypropylene tubes, and immediately stored at -70°C for subsequent analysis. In addition, whole blood was processed within 2 hrs of sample collection, transferred into polypropylene tubes, and stored at -20°C until further use.

### Sputum induction and analysis

According to the method described by Spanevello *et al.*, the sputum was induced through inhalation of hypertonic saline solution (4.5%) with an ultrasonic nebulizer (DeVilbiss 65; DeVilbiss Corporation, Somerset, PA) and analysed after plug selection [37].

### *Measurement of *LTB-4 and IL-8

A specific enzyme immunoassay (Cayman Chemical, Ann Arbor, MI and Thermoscientific, Rockford, IL, USA) was used to measure LTB-4 and IL-8 in breath condensate and whole blood. The intra-assay and inter-assay variability were less than 10%. The specificity was 100%, and the detection limits of the assay were 3 pg/ml and 2 pg/ml. We tested the reproducibility of the repeated measurements of LTB-4 and IL-8 by means of the Bland and Altman test and with the variation coefficient [38].

### Statistical analysis

The data were not normally distributed and therefore non-parametric tests were chosen for analysis. The data were expressed as a median (25% percentile-75% percentile). A Mann-Whitney test was used to compare the groups, and the correlations between variables were performed using the Spearman's rank correlation test. Significance was defined as a p value of < 0.05.

Statistical analysis was carried out using a SATA 10 MP for MAC OS × software package. The data were analyzed by the Department of Medical Sciences, Section of Hygiene, University of Foggia and Apulia Regional Epidemiological Observatory, Italy.

## Results

### LTB-4 *measurement*

**Figure 1:**
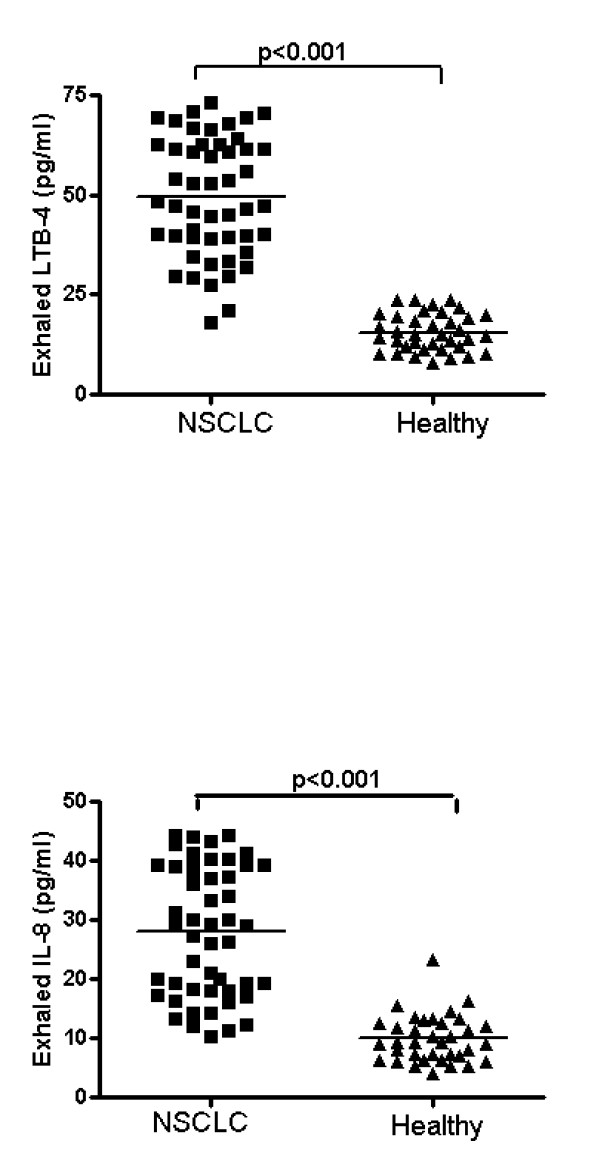
**LTB-4 (Panel A) and IL-8 (Panel B) levels were significantly higher in the EBC of NSCLC patients than in the EBC of control subjects (p < 0.001)**.

**Figure 2:**
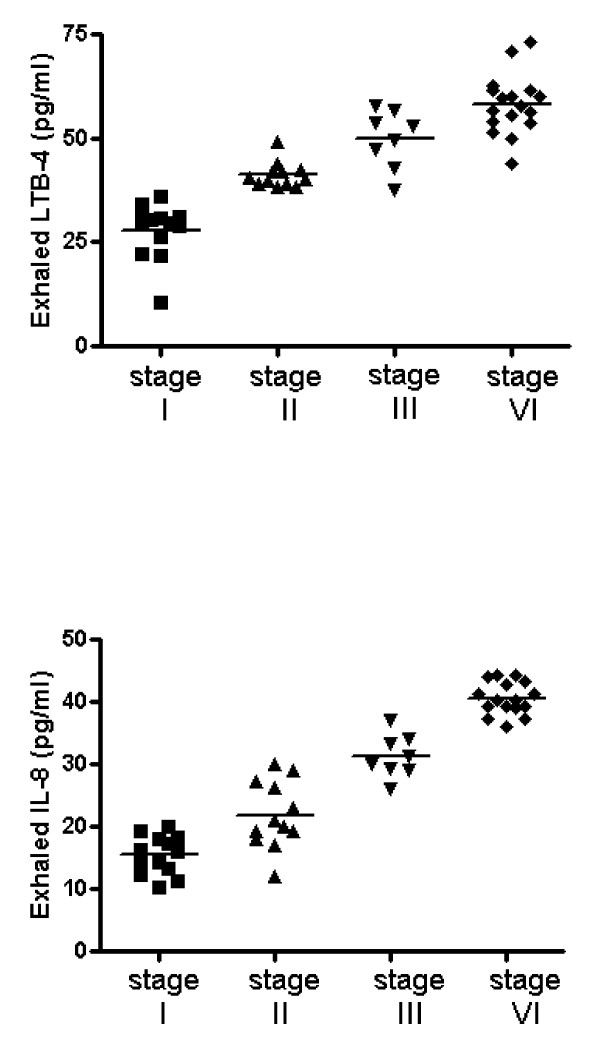
**Progressively higher concentrations of LTB-4 (Panel A) and IL-8 (Panel B) in EBC were found from stage I to stage IV**. Significant differences between each stage and the next one were observed (p < 0.005).

LTB-4 levels were significantly higher in the EBC of NSCLC patients than in the EBC of control subjects [43.5 (35.1-56.5) vs 15.9 (7.9-23.5) pg/ml, p < 0.001] (Figure 1, panel A). Significantly higher concentrations of LTB-4 were observed in the whole blood of NSCLC patients than in controls [44.1 (21.7-69.5) vs 17.9 (13.4-31.2) pg/ml, p < 0.001]. Progressively higher concentrations of LTB-4 in EBC were found from stage I to stage IV [29.8 (24.2-31.2), 40.3 (39.0-42.2), 51.2 (45.2-55.2) and 57.9 (53.8-61.5) pg/ml] (Figure 2, panel A). Significant differences between each stage and the next one were observed (p < 0.005).

No difference in exhaled LTB-4 levels was reported among the histological types of NSCLC.

**Figure 3:**
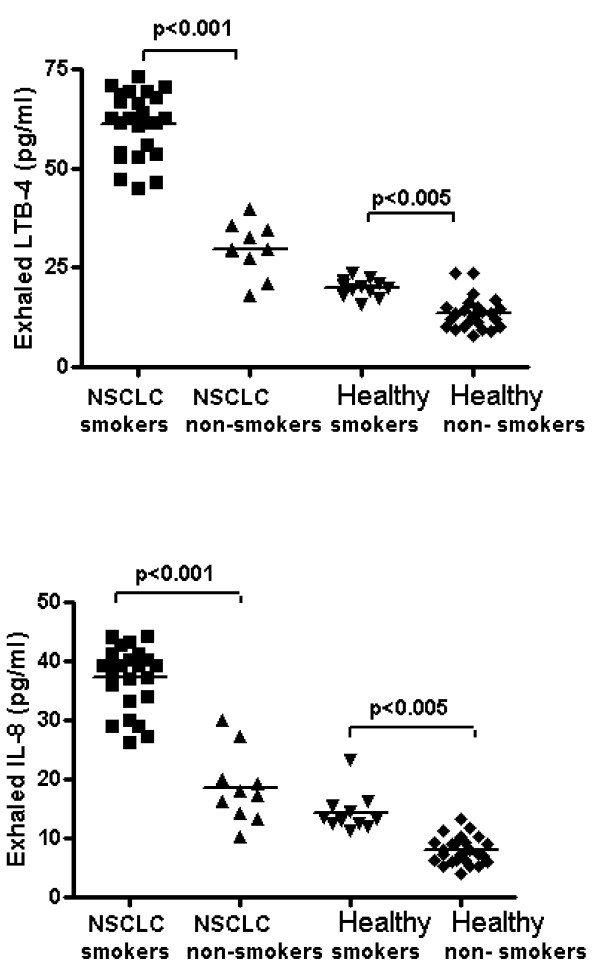
**LTB-4 (Panel A) and IL-8 (Panel B) levels were significantly higher in the EBC of smokers compared to non smokers either in NSCLC or in healthy group (p < 0.001, p < 0.005)**.

Higher levels of exhaled LTB-4 were found in smokers than in non-smokers both in the case of NSCLC patients and controls [47.9(18.9-62.8) vs 33.4 (17.9-48.3) pg/ml, p < 0.001 and 18.4 (13.5-23.5) vs 12.3 (7.9-16.8) pg/ml, p < 0.005)] (Figure 3, panel A). Increased levels of exhaled LTB-4 were observed in group 2 with respect to group 1 in NSCLC patients [60.6 (44.3-62.8) vs 35.3 (18.9-44.3) pg/ml, p < 0.001]. A positive correlation was found between LTB-4 concentrations in EBC and neutrophils in the induced sputum (r = 0.7, p < 0.001).

No correlation was observed between LTB-4 concentrations in EBC and other clinical and demographic data.

### IL-8 *measurement*

IL-8 levels were significantly higher in EBC of NSCLC patients than in EBC of control subjects [28.1 (10.3-44.2) vs 10.7 (4.1-23.4) pg/ml, p < 0.001] (Figure 1, panel B). Significantly higher concentrations of IL-8 were observed in whole blood of NSCLC patients than in controls [38.7 (27.1-50.2) vs 16.4 (10.2-22.6) pg/ml, p < 0.001]. Progressively higher concentrations of IL-8 in EBC were found from stage I to stage IV [15.5 (10.3-20.1), 21.9 (12.3-30.1), 31.3 (26.1-37.1) and 40.5 (36.1-44.2) pg/ml] (Figure 2, panel B). Significant differences between each stage and the next one were observed (p < 0.005).

No difference in exhaled IL-8 levels was reported between the histological types of NSCLC.

Higher levels of exhaled IL-8 were found in smokers than in non-smokers both in NSCLC patients and in controls [38.3(30.6-44.2) vs 19.4 (10.3-30.1) pg/ml, p < 0.001 and 13.2 (13.5-23.4) vs 7.3 (4.1-13.4) pg/ml, p < 0.005] (Figure 3, panel B). Increased levels of exhaled IL-8 were observed in group 2 with respect to group 1 in NSCLC patients [40.3 (37.8-44.2) vs 32.3 (27.1-40.2) pg/ml, p < 0.001]. A positive correlation was observed between IL-8 concentrations in EBC and neutrophils in the induced sputum (r = 0.7, p < 0.001).

No correlation was observed between IL-8 concentrations in EBC and other clinical or demographic data.

### Cytology in the induced sputum

Twenty NSCLC patients and twenty-five healthy subjects were not able to produce adequate sputum samples (defined as containing at least 500 non-squamous cells) and their expectorates were discharged.

The percentage of neutrophils was higher in the NSCLC patients than in the controls [32.3 (27.5-36.2) vs 10.4 (8.3-13.7); p < 0.01]. The percentage of macrophages was lower in the NSCLC patients than in the controls [63.4 (55.1-71.6) vs 85.8 (78.4-87.9); p < 0.01], whereas no significant differences were observed in the percentage of eosinophils [1.2 (0.9-2.0) vs 1.2 0.8-2.0)], lymphocytes [1.0 (0.3-2.0) vs 1.0 (0.8-1.9)] and epithelial cells [OO: 0.9 (0.6-1.7) vs 0.9 (0.6-1.9)].

## Discussion

The concentrations of LTB-4 and IL-8 explored by the present study were found to be higher in the exhaled breath condensate and whole blood of NSCLC patients compared to controls, particularly in smokers and ex-smokers rather than in non-smokers. An overproduction of LTB-4 and IL-8 in the EBC of subjects with more advanced stages of lung cancer and a relation to cancer progression were further described. Finally, we reported a positive correlation between exhaled LTB-4 and IL-8 and the percentage of neutrophils in the sputum.

One recent intriguing theory on lung cancer pathogenesis supposed that chronic inflammation can promote an environment that is conducive to carcinogenesis. Neutrophils seem to be the main orchestrators, fighting against cancers by eradicating dysplastic and neoplastic cells. Moreover, these inflammatory cells can be manipulated to induce an immune escape of cancer cells, especially in a tumour-promoting micro-environment, which is created by a chronic inflammation seen in lungs [20]. Furthermore, repeated lung injury and repair triggered by chronic inflammation enhance cell turnover and potential genetic error, as well as epithelial-to-mesenchymal transition and ultimately lead to lung tumorigenesis [39]. Regrettably, notwithstanding the recent increasing interest in the neutrophilic inflammatory origin of lung cancer, the role of inflammation and immunity in tumour biology remains complex and not completely known.

In order to give a contribution to this curious inflammatory origin of lung tumour, we studied two known neutrophilic inflammatory proteins, namely LTB-4 and IL-8, in the airways and blood of patients affected by lung cancer.

Several previous studies analysed these proteins in lung cancer patients and described high levels of LTB-4 and IL-8 in tumour tissue [31], pleural effusion [19,22], BAL [30] and serum [28-30].

In accordance with previous results, we confirmed the increase of LTB-4 and IL-8 in the systemic compartment and in the airways, reporting higher concentrations in the whole-blood and breath condensate of NSCLC patients compared to controls.

Both the LTB-4 and the IL-8 have been previously measured as neutrophilic markers in the breath condensate of patients affected by lung inflammatory diseases [40-43]. In the light of the trendiness of studies of tumour markers in the exhaled breath condensate, for the complete non-invasiveness of this collection method, which is known to be well accepted by patients and ethic committees, IL-8 was also recently measured in the breath condensate of NSCLC patients. In this regard, however, contrasting data are available: in one study the exhaled IL-8 did not result as indicative of lung cancer [32], while in another its levels decreased after two cycles of chemotherapy [28]. In accordance with the Jungraithmayr study, we observed that exhaled IL-8 reflects cancer biology as its concentrations increased in lung cancer patients compared to controls, particularly when progressing to the next stage of cancer. We reported the same behaviour also for LTB-4, which we measured in this case for the first time in the breath condensate of lung cancer patients.

Our findings on the role of LTB-4 and IL-8 in the progression of lung tumours were not surprising in consideration of the fact that previous studies indicated that these proteins possess angiogenic and direct mitogenic effects. Higher concentrations of both markers were in fact largely described in loco regional relapse as well as the metastasis of lung cancer [20,23-26,44].

Notwithstanding the numerous studies, none reported differences in the LTB-4 and IL-8 levels in adenocarcinoma and squamous cell carcinoma. Moreover, in this study we did not observe any correlation with any hystopathological type as similar concentrations of exhaled LTB-4 and IL-8 were found in both hystotypes.

Another interesting result of this study was the higher concentration of IL-8 and LTB-4 that we described in smokers rather than in non-smokers. This is perfectly in line with what was suggested by Pickett [45], who showed changes in cytokine production in primary-normal human bronchial epithelial cells following treatment for 18 hrs with cigarette smoke condensates. The increase of these proteins in smokers suggests that the increased inflammatory cells in airways, largely described in smokers, are another possible source of these proteins. In detail, the polymorphonuclear cells seem to be involved in the production and release of LTB-4 and IL-8 [15-18] as was also proved by the positive correlation that we reported between these exhaled proteins and the percentage of neutrophils in the sputum. However, as previously suggested by Petrin and Fudala, this correlation may also be the result of the chemoactractant or pro-survival properties of these mediators [46,47]. In this regard, the study of Pace et al demonstrated [19] that LTB4 is an indicator of the state of activation of neutrophils [19] as well as stimulating peripheral blood neutrophils to synthesize and secrete biologically active IL-8 [48].

Although healthy smokers showed higher levels of exhaled IL8 and LTB4 compared to non-smokers, the concentrations of both markers were significantly higher in NSCLC patients (smokers and non-smokers), allowing us to suggest that their increase is not only due to cigarette smoke but is also the effect of lung cancer.

In this study we support and encourage the use of the breath condensate in lung cancer studies in the light of the non-invasiveness of this method that, especially in lung cancer patients, where possible, is auspicable in view of the emotional and practical stress that patients underwent.

## Conclusion

In this study we support the key role of neutrophilic airways inflammation in lung carcinogenesis and highlight the potentiality of LTB-4 and IL-8 as markers of lung cancer development and progression [30,31,39].

This said, although further studies are needed to confirm our data, we believe that the measurement of LTB-4 and IL-8 in breath condensate could soon be proposed in the diagnosis and monitoring of NSCLC. This can be justified when one bears in mind the non-invasiveness of this method, not to mention the fact that it lends itself particularly well to the early screening and follow-up of lung cancer.

## Conflict of interest

All the authors disclose any financial, personal or other relationships with other people or organizations. They did not receive any source of funding or writing assistance.

## Authors' contributions

GEC conceived the study, participated in its design and co-ordination and wrote the manuscript, whereas GPP carried out the markers dosage and DL performed the statistical analysis.

AK and SO enrolled patients, whereas MPFB participated in the revision of manuscript. All the authors both read and approved the final manuscript.

## Pre-publication history

The pre-publication history for this paper can be accessed here:

http://www.biomedcentral.com/1471-2407/11/226/prepub
